# ^15^N NMR Shifts of Eumelanin Building Blocks in Water: A Combined Quantum Mechanics/Statistical Mechanics Approach

**DOI:** 10.3390/molecules25163616

**Published:** 2020-08-09

**Authors:** Leonardo Bruno Assis Oliveira, Tertius L. Fonseca, Benedito J. C. Cabral

**Affiliations:** 1 Departamento de Física, CEPAE, Universidade Federal de Goiás, Goiânia 74690-900, GO, Brazil; leonardobruno@ufg.br; 2 Instituto de Física da Universidade Federal de Goiás, Goiânia 74690-900, GO, Brazil; tertius@ufg.br; 3 BioISI-Biosystems and Integrative Sciences Institute, Faculty of Sciences, University of Lisboa, 1749-016 Lisboa, Portugal

**Keywords:** eumelanin, photoprotective pigments, ^15^N NMR shielding constants, solvent effects, π stacking, NMR

## Abstract

Theoretical results for the magnetic shielding of protonated and unprotonated nitrogens of eumelanin building blocks including monomers, dimers, and tetramers in gas phase and water are presented. The magnetic property in water was determined by carrying out Monte Carlo statistical mechanics sampling combined with quantum mechanics calculations based on the gauge-including atomic orbitals approach. The results show that the environment polarization can have a marked effect on nitrogen magnetic shieldings, especially for the unprotonated nitrogens. Large contrasts of the oligomerization effect on magnetic shielding show a clear distinction between eumelanin building blocks in solution, which could be detected in nuclear magnetic resonance experiments. Calculations for a π-stacked structure defined by the dimer of a tetrameric building block indicate that unprotonated N atoms are significantly deshielded upon π stacking, whereas protonated N atoms are slightly shielded. The results stress the interest of NMR experiments for a better understanding of the eumelanin complex structure.

## 1. Introduction

Melanins are a class of biopolymers derived from the tyrosine oxidation process, which make up the human pigment system, have antioxidant properties and can act as free radical scavengers (in particular, metal ions from oxidation-reduction processes), preventing cells against the oxidation process [1,2,3]. There are two main classes of melanins: eumelanins, which give brown-black colors and pheomelanins, which favor yellow-red tones. Their main functions are associated with skin, hair and iris pigmentation and photoprotection [4,5]. But they can also play other important biological functions in degenerative diseases like the Parkinson’s disease (neurodegenerative disorder) [6,7] and age-related macular degeneration [8]. Melanins also exhibit electrical conductivity and photoconductivity in the condensed phase [9,10,11].

Despite the great interest and intense study on melanins in recent decades [12,13], the structure and definitive composition of eumelanins (the most prevalent and important form of melanins) are not yet fully known. Eumelanins comprise molecular structures derived from the coupling of 5,6-dihydroxyindole (DHI) and 5,6-dihydroxyindole-2-carboxilic acid (DHICA) monomers [14,15], with structural morphology observed in images of atomic force microscopy (AFM) that reflect aggregation of different oligomeric species [16]. Two main molecular models have been proposed to explain the structure of eumelanin pigments: one considers that eumelanin is an extended polymer system [17,18,19,20]; the other considers that eumelanin is composed of smaller oligomeric structures consisting of DHI, DHICA, and the redox and tautomeric species, indolequinone (IQ) and quinone methide (MQ) [21,22]. Additional support for the finite size molecular model of eumelanin pigments has been reported for synthetic eumelanin by using scanning tunneling microscopy (STM) [22], small-angle neutron scattering (SANS) [23,24], and small-angle X-ray scattering (SAXS) data [24]. Experimental NMR studies on poly(dopamine), a synthetic form of melanin, pointed out the presence of noncovalent supramolecular aggregates stabilized by π stacking, charge transfer, and hydrogen bonding [25]. The presence of aggregates stabilized by hydrogen bonding has been, however, questioned by other experimental work based on different methods including NMR spectroscopy [26]. In addition, Lorite et al. [27] have shown based on AFM images of synthetic eumelanin films prepared in both aqueous and organic solvents that the solvent interactions may drive important structural features; films can exhibit relatively planar structures in solution, but the aggregation process of synthetic melanin is enhanced in aqueous solution.

Regarding that water in the biological medium is always a major component, it is expected that the presence of water in the vicinity of the of eumelanin building blocks affect their electronic properties. Previous theoretical works [28,29,30,31] have pointed out the importance of the inclusion of solvent effects on the electronic spectra of melanin building blocks based on a simplified representation of the environment by a dielectric continuum. In protic solvents, however, the structural and electronic properties of eumelanin pigments can be particularly affected by electrostatic interactions, and the role played by hydrogen bonding and long-range polarization effects in the spectroscopic properties of monomeric and oligomeric building blocks certainly deserve special attention. Indeed, we have shown that for some eumelanin building blocks in liquid water, the light absorption at higher energies is enhanced as a result of the long-range electrostatic interactions with the water environment [32]. It is also shown that the broadening of electronic absorption spectrum with a nearly monotonic decay from the ultraviolet to the near infrared can be obtained by the superposition of the electronic spectra of different monomeric and oligomeric building blocks, in line with the predictions of the chemical disorder model proposed to explain the eumelanin absorption spectrum [33,34]. More recently, Nogueira et al. [35] have shown, based on a theoretical-experimental investigation, that in the excited-state deactivation mechanism of a eumelanin building block in water and in methanol occurs through a reorganization of the explicit solvent molecules around eumelanin monomer to assist a sequential proton-coupled electron transfer process.

In the present work, we study the magnetic shielding constant σ(${}^{15}$N) of a selected set of eumelanin building blocks including monomers, dimers, and tetramers in aqueous solution using a sequential quantum mechanics/molecular mechanics (S-QM/MM) method [36,37,38] in combination with the average solvent electrostatic configuration (ASEC) model [39]. The structures of the liquid composed by the solute and solvent molecules in the thermodynamic equilibrium were generated using MM simulations whereas the QM calculations were performed using the density functional theory (DFT) that offer a good compromise between computational cost and accuracy for molecular magnetic properties in condensed phase [40,41,42]. Previous studies have been reported showing the importance of the analysis of electronic properties of building blocks for a better understanding of spectroscopic properties of eumelanin pigments [3,29,43,44]. It should be stressed that, over time, nuclear magnetic resonance experiments are often made in solution and consideration of the liquid environment imposes analysis of the statistical nature that is inherent to liquids. Thus, the appropriate theoretical treatment of the solvent effects must include the specific thermodynamic condition. Specifically, the role played by hydrogen bonding [45,46] on NMR properties in solution deserves special attention.

## 2. Theoretical Methods

**Figure 1:**
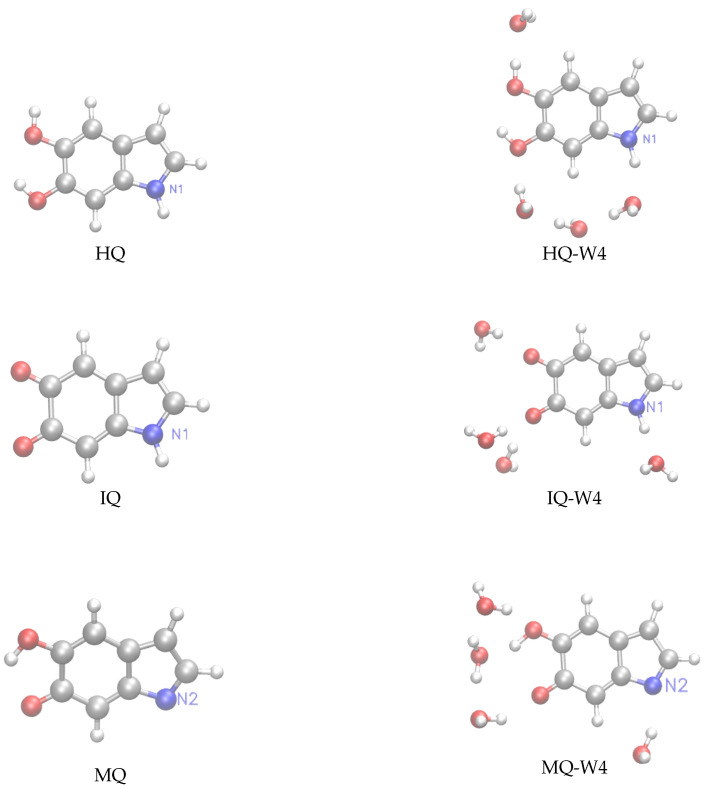
**Left panels:** structure of the hydroquinone (HQ), indolequinone (IQ), and quinone methide (MQ) monomers. **Right panels:** microsolvated structures with for water molecules HQ-W${}_{4}$, IQ-W${}_{4}$, and MQ-W${}_{4}$.

**Figure 2:**
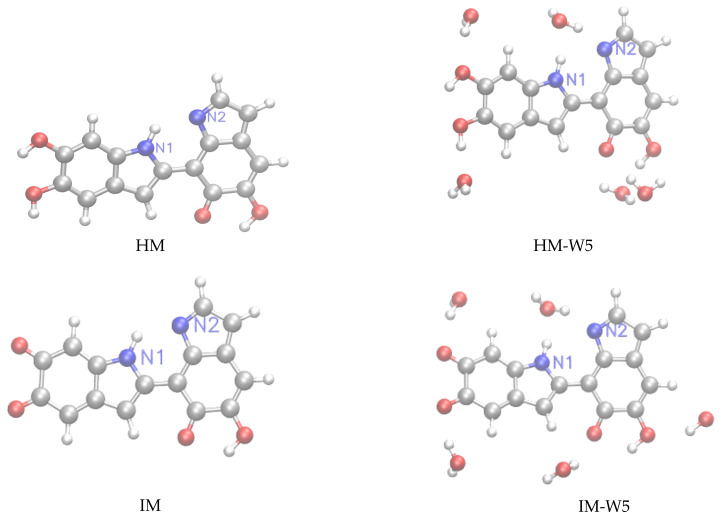
**Left panels:** structure of the HM≡HQ+MQ and IM≡IQ+MQ dimers. **Right panels:** microsolvated structures with five water molecules (HM-W5, IM-W5).

**Figure 3:**
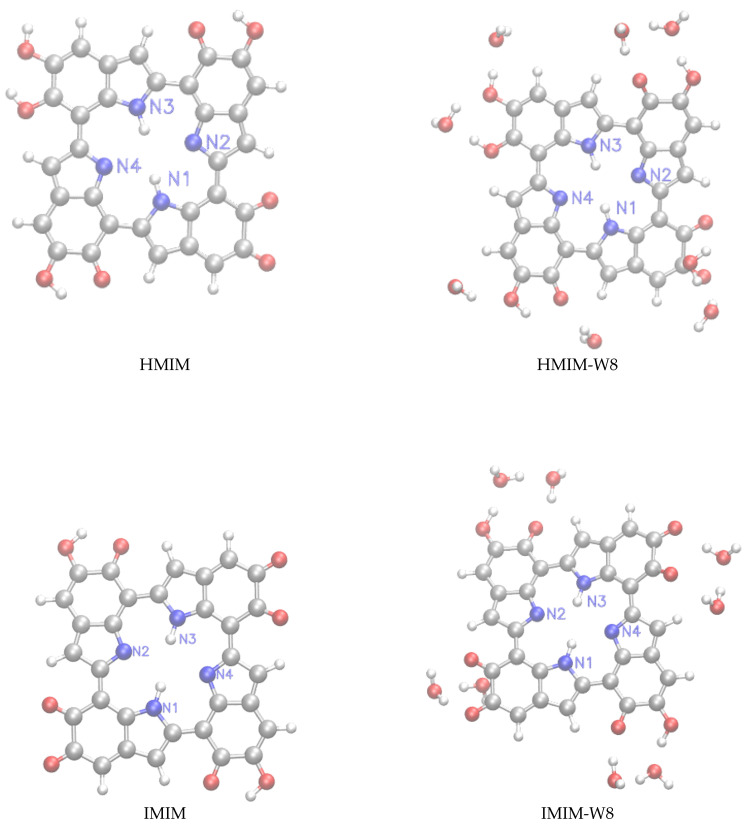
**Left panels:** structure of the HMIM≡HM+IM and IMIM≡IM+IM tetramers. **Right panels:** microsolvated structures with eight molecules (HMIM-W${}_{8}$, IMIM-W${}_{8}$).

Following our previous work [32], we have considered for this study monomeric structures composed of fundamental building blocks including DHI (5,6-dihydroxyindole) or hydroquinone (HQ) and the redox and tautomeric species, indolequinone (IQ) and quinone methide (MQ). Two dimers defined by covalent bonding of HQ and MQ (named HM) and of IQ and MQ (named IM). And two tetrameric structures proposed by Kaxiras et al. [47] formed by the dimers HQ and IM (HMIM) and IM and IM (IMIM), in arrangements that contain an inner porphyrin ring. It is also considered micro-solvated structures of eumelanin building blocks with selected water molecules in close hydrogen bond (HB) interaction. Thus, the complexes of DHI or HQ, IQ and MQ monomers with four water molecules (HQ-W4, IQ-W4 and MQ-W4), of HM and IM dimers with five water molecules (HM-W5 and IM-W5) and of HMIM and IMIM tetramers with eight water molecules (HMIM-W8 and IMIM-W8) were investigated. The isolated optimized structures for the monomers, dimers, and tetramers and the corresponding microsolvated structures are shown in Figure 1, Figure 2, and Figure 3, respectively.

The geometry of these of isolated and micro-solvated monomeric and oligomeric building blocks were fully optimized with the hybrid B3LYP exchange-correlation functional [48]. Dunning’s correlation consistent cc-pVxZ (x = D,T) basis sets [49] were used in these calculations. Geometry optimizations of the isolated building blocks (monomers and dimers) were carried out with the cc-pVTZ basis-set. A cc-pVDZ basis set was used for tetramers, the IMIM dimer, and microsolvated structures. Solvent effects due to the water environment were taken into account by using different models. The first one is a microsolvation approach in which the solute is a melanin building block in interaction with a few water molecules (MS-W${}_{n}$), where *n* is the number of water molecules. In the second model, we add to MS-W${}_{n}$ a self-consistent reaction field (SCRF) where the water environment is represented by a continuum dieletric medium. In the present calculations SCRF corresponds to the polarizable continuum model (PCM) [50]. This model is named MS-W${}_{n}$+PCM.

The third approach for including solvent effects relies on the ASEC model, where the water molecules are represented by point charges defining an electrostatic embedding. The ASEC model was applied, sequentially, to selected configurations generated by Monte Carlo. For the monomers (HQ, IQ, and MQ), a model named HB+PC, which is based on configurations generated by MC was also exploited. In the HB+PC model, for each selected MC configuration, a quantum system including the solute and the first solvation shell is defined, whereas the remaining water molecules are treated as simple point charges centered at the atomic sites. Monte Carlo (MC) simulations of eumelanin building blocks were carried out with the DICE program [51] in the NpT ensemble at a temperature T=300 K and pressure p=1 atm for systems with one solute molecule (HQ, IQ, and MQ monomers, and HM and IM dimers) and 1001 water molecules. The MC simulations for the HMIM and IMIM tetramers in water were carried out with 2500 solvent molecules. Intermolecular interactions were modeled by the standard Lennard–Jones (LJ) and Coulomb potential with three parameters for each interacting site. For water the TIP3P model from [52] was used as force field. For the different solutes, the LJ parameters were defined by the liquid simulation (OPLS) force field [53] and the atomic charges were obtained using an electrostatic potential fit (CHELPG) [54]. The geometry of each eumelanin building block in water was optimized using the polarizable continuum model (PCM) [50] and was kept as rigid geometry during MC simulations. Effects of the electronic polarization of solute by the environment were included using a reliable iterative scheme, as reported in previous works [55,56,57].

In the QM calculations of the magnetic shielding constant σ(${}^{15}$N) of the eumelanin building blocks in liquid water, we have used the gauge including atomic orbitals (GIAO) approach to ensure gauge-origin invariant results [58,59]. The magnetic shieldings were calculated using GIAO at the B3LYP/6-311++G(2d,2p) level, as implemented in the GAUSSIAN-09 [60] program. In order to obtain statistically converged results for this electronic property, we have selected 400 uncorrelated configurations to generate the average solvent electrostatic configuration (ASEC) [39], where the solvent molecules are treated as simple point charges that define an electrostatic embedding. The charges of this single configuration were then normalized by the number of selected configurations. In the present case, the atomic charges of 40,000 solvent molecules (400 water molecules × 100 configurations) were included in the ASEC configuration. Both geometry optimization and properties calculations were performed using the GAUSSIAN-09 [60] program.

## 3. Results and Discussion

### 3.1. Monomers

In a first analysis of the hydration effect, we consider micro-solvated structures of monomers, dimers and tetramers with explicit water molecules in close hydrogen bond interaction. The number of water molecules in each micro-solvated structure varies according to the size of the eumelanin building blocks (monomers, dimers, and tetramers) and different possibilities of making hydrogen bonds should be taken into account. B3LYP/6-311++G(2d,2p) results for the ${}^{15}$N shielding constants of HQ, IQ e MQ monomers in gas phase and water are quoted in Table 1.

In gas phase, there is a shielding effect for the protonated nitrogen (N1 in HQ and N1 in IQ) while the unprotonated one (N2 in MQ) is deshielded. For the micro-solvated structures of HQ and IQ with four explicit neighboring solvent water molecules, the results obtained with the MS-W${}_{4}$ model indicate that protonated nitrogens are deshielded, with reductions in the $\sigma_{\mathrm{HQ}}$(${}^{15}$N1) and $\sigma_{\mathrm{IQ}}$(${}^{15}$N1) values of −18.40 and −23.45 ppm, respectively, in relation to the gas phase results. Comparison between MS-W${}_{4}$ and MS-W${}_{4}$+PCM indicates that polarization effects due to the water environment lead to a further deshielding of the protonated N1 (HQ and IQ) and to a shielding of the unprotonated N2 (MQ). The results for protonated N1 from the MS-W${}_{4}$+PCM model are closer to the values predicted by HB+PC than those from ASEC. We notice that the main differences between MS-W${}_{4}$+PCM are the representation of long-range polarization effects by a continuum dielectric medium (PCM response), as compared to polarization effects due to point charges and the inclusion of thermal effects (HB+PC).

In liquid phase, the ASEC model gives a smaller deshielding effect, whose corresponding reductions are −8.09 and −16.59 ppm. It is interesting to note that the oxidation of HQ to IQ affects the nitrogen magnetic shieldings. However, the difference between the ASEC results for $\sigma_{\mathrm{HQ}}$(${}^{15}$N1) and $\sigma_{\mathrm{IQ}}$(${}^{15}$N1) induced by oxidation decreases in solution (2.22 ppm), when compared to the gas phase result (10.77 ppm). In the opposite direction, the hydration effects lead to a shielding effect for $\sigma_{\mathrm{MQ}}$(${}^{15}$N2) with increases of 20.83 ppm in the micro-solvated situation (MS-W${}_{4}$ model) and of 36.74 ppm in the liquid one (ASEC model), relative to the gas phase results.

**Figure 4:**
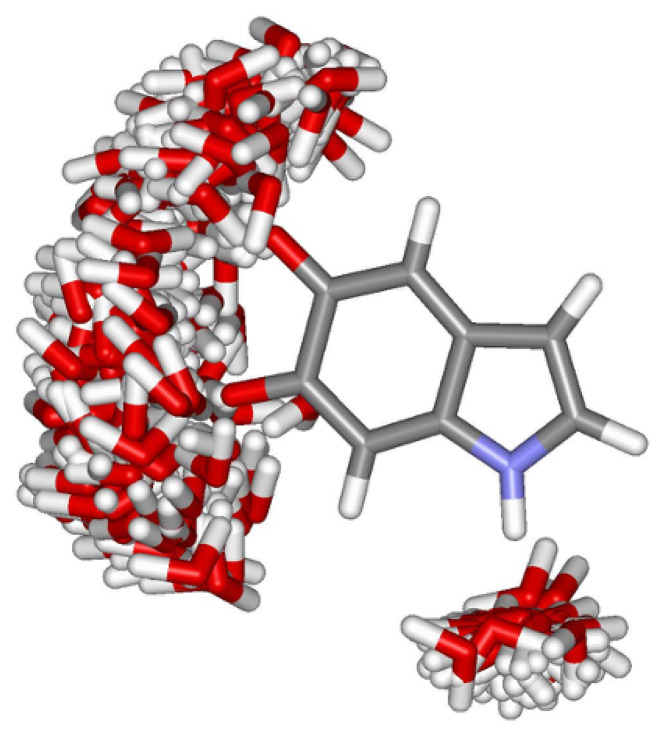
Hydrogen bond configuration space obtained for IQ in aqueous solution. The figure corresponds to the superposition of 100 statistically uncorrelated configurations obtained from classical Monte Carlo simulations. Oxygen (red), hydrogen (white), nitrogen (blue) and carbon (grey).

Although the micro-solvated structures provide an estimate of the effect of the hydrogen bonds on the nitrogen magnetic shieldings, a proper description of the specific short-range interactions with the environment requires that the thermal disorder of the hydrogen-bonded water molecules be taken into account. Thus, we have selected hydrogen-bonded structures from MC simulations and used them in the QM calculations to obtain a more realistic description of the solvent effects on the magnetic properties of the monomeric species. Because the monomeric species are relatively stiff, they are kept with rigid geometry during the QM calculations of $\sigma {(}^{15}N)$ for the hydrogen-bonded structures generated by the Monte Carlo simulations. This means that vibrational corrections to the isotropic shielding constants are neglected (for $\sigma {(}^{15}N)$ they correspond typically to ∼−2.3% [61]). However, vibrational corrections to the chemical shift are expected to be significantly smaller because, for a given atom, they are related to the difference between two small corrections. In the ASEC model thermal effects on the shielding constants are related to the Monte Carlo sampling of the configurations. Figure 4 illustrates the configuration space occupied by all the water molecules that are hydrogen bonded to IQ. This allows a clear visualization of the configuration space occupied by the hydrogen bonded water molecules.

As reported in a previous work [32], on average 3.5 water molecules are hydrogen bonded to HQ. For IQ and MQ these numbers are 4.7 and 3.5, respectively. The number of HBs to nitrogen atoms for the monomeric species is around 1. A detailed analysis of the structure of hydrogen bonds for oligomers of eumelanin building blocks considered here in water is presented in Ref. [32].

We noticed from results of Table 1 that the thermal disorder effect due to the inclusion of explicit water molecules could significantly affect the nitrogen magnetic shieldings. For N1 in HQ and IQ, that act as proton donors, the HB+PC model gave equivalent results for σ(${}^{15}$N) of 99.99 and 99.97 ppm, with noticeable solvent shifts of −14.56 and −25.30 ppm. In comparison with the ASEC results, the presence of explicit water molecules that made hydrogen bonds gave a deshielding effect, with reductions for $\sigma_{\mathrm{HQ}}$(${}^{15}$N1) of −6.47 ppm and for $\sigma_{\mathrm{IQ}}$(${}^{15}$N1) of −8.71 ppm. For comparison, significant solvent shifts have also been reported for the magnetic shielding of protonated nitrogens of nucleic acids in liquid water due to the hydrogen bond effects [42]. Note that, in contrast with the gas phase results, similar magnetic shieldings of the protonated nitrogens indicated that an ${}^{15}$N NMR measurement could not distinguish the HQ and IQ monomers in water, whereas the ASEC one gave a difference of 2.22 ppm. For N2 in MQ, that act as proton acceptor, the HB+PC model predicted for $\sigma_{\mathrm{MQ}}$ (${}^{15}$N1) a result of −52.23 ppm, with a marked solvent shift of 34.32 ppm. N2 was deshielded in −2.42 ppm due to the presence of explicit water molecules, in comparison with the ASEC result. Comparison between HB+PC and MS-W${}_{4}$ results showed that protonated nitrogen magnetic shieldings were little affected by the thermal disorder effect, with differences of −3.84 and 1.85 ppm for N1 in HQ and N1 in IQ, respectively. The effect of the thermal disorder was larger for the unprotonated nitrogen magnetic shielding, and the corresponding difference when we compared MS-W4 and HB+PC models was of 13.49 ppm for N2 in MQ. A smaller difference (4.45 ppm) was observed when we compare MS-W${}_{4}$+PCM and HB+PC models. In this case, in addition to thermal effects, the difference reflected long-range polarization effects that are taken into account in MS-W${}_{4}$+PCM. This gives an indication of the order of magnitude of the changes in the magnetic shieldings of nitrogen atoms in micro-solvated structures that are directly involved in hydrogen bonds in the aqueous environment.

### 3.2. Dimers and Tetramers

The previous calculations show that for a proper description of both nitrogen magnetic shieldings of the monomers in water it is important to include the surrounding molecules in the treatment of the solvation effects. Therefore, the number of HBs through N atoms is significantly reduced (close to zero) for HM and IM dimers and HMIM and IMIM tetramers in water [32]. Here, we present a first estimation of the changes in the nitrogen shielding of dimers and tetramers due to the hydrogen bond effects based on calculations of micro-solvated structures.

Table 2 shows the B3LYP/6-311++G(2d, 2p) results for the ${}^{15}$N magnetic shielding constants of the HM and IM dimers in gas phase and water.

For HM and IM with five water molecules (HM-W${}_{5}$ and IM-W${}_{5}$), the MS-W${}_{5}$ model gives a deshielding effect for protonated nitrogen magnetic shieldings, with changes, respectively, of −6.27 and −10.10 ppm, relative to the gas phase results. The corresponding changes, for unprotonated nitrogen magnetic ones, are 4.56 and 1.12 ppm. Comparison between MS-W${}_{5}$ and MS-W${}_{5}$+PCM show similar trends concerning shielding and deshielding of the N atoms. We notice that the optimized structures of HM-W${}_{5}$ and IM-W${}_{5}$ represented in Figure 2 show similar hydrogen bond interactions with N1 and N2. It is reasonable to assume that different optimized configurations will change the N shielding constants. This is one of the well known limitations of the microsolvation approach, namely, the dependence of the results on the specific optimized structures, which is in contrast with the sampling of representative configurations generated by Monte Carlo that is used, for example, in the ASEC method. From ASEC the protonated N1 atoms are deshielded by −0.10 ppm (HM) and −8.49 ppm (IM), whereas unprotonated N2 are shielded by 5.28 ppm (HM) and 4.52 ppm (IM).

Table 3 shows the B3LYP/6-311++G(2d, 2p) results for the ${}^{15}$N magnetic shielding constants of the HMIM and IMIM tetramers in gas phase and water.

For the HMIM and IMIM tetramers with eight water molecules, the magnetic shieldings of the inner nitrogen atoms should be much less affected by hydrogen bonding than in the case of monomers and dimers. Therefore, it would be expected that a correct description of long-range polarization effects should be more important than the explicit inclusion of local hydrogen bond interactions. For HMIM-W${}_{8}$, MS-W${}_{8}$+PCM results show that N1 and N3 atoms were deshielded by −0.54 and −3.12 ppm, respectively, whereas N2 and N4 were now shielded by 4.53 and 0.97 ppm. Interestingly, MS-W${}_{8}$+PCM predicted that for IMIM-W${}_{8}$, both protonated and unprotonated N atoms were slightly deshielded. We noticed, however, that the dipole moments of HMIM-W${}_{8}$ and IMIM-W${}_{8}$ were 13.9 and 1.49 D, respectively. Therefore, the dielectric reaction field from the water environment for the more dipolar species (HMIM) could be a possible explanation for the different behaviour of the unprotonated N magnetic shielding in these two eumelanin building blocks.

For HMIM, the ASEC model predicted negligible solvent effect on the magnetic shielding of the protonated N1 (1.1 ppm) and N3 (−0.18 ppm) atoms. This was a general trend, and the only exception was the shielding by 9.1 ppm of the unprotonated N4 atom of HMIM. ASEC results relied on an average single electrostatic configuration. This configuration was determined from a set of MC configurations, generated by a classical interaction potential, and where local hydrogen bonding was only implicitly included in a classical way. In addition, the presence of a true three dimensional solvation framework in ASEC made, in this case, comparison with the MS-W${}_{8}$ + PCM model difficult.

One can see that the magnetic shielding of the nitrogen atoms in the tetramers (see Table 3) was much less affected by the solvent effect than the magnetic ones of the monomers (see Table 1). For HM and IM dimers, the ASEC model predicted protonated nitrogens deshielded, with solvent shifts of −0.10 and −8.49 ppm, respectively, although the solvent effect for N1 in HM was negligible.

For N2 in HM and IM, the corresponding solvent shifts were 5.28 and 4.52 ppm. Similarly, the protonated nitrogens in tetramers, N1 and N3 in HMIM and in IMIM, were slightly deshielded in water, and the ASEC model gave a solvent shift range of −2.41 to 4.75 ppm (Table 3). In this case, N3 in IMIM was shielded. For the unprotonated ones, N2 and N4 in HMIM and IMIM, the corresponding solvent shift range was of −3.32 to 9.1 ppm, being N4 in HMIM shielded.

It was also observed, in contrast with the monomeric units, that the oxidation of HM to IM [HMIM to IMIM] had a significant effect on protonated [unprotonated] nitrogens magnetic shieldings. For instance, the difference between the ASEC results for $\sigma_{\mathrm{HM}}$(${}^{15}$N1) and $\sigma_{\mathrm{IM}}$(${}^{15}$N1) [$\sigma_{\mathrm{HMIM}}$(${}^{15}$N2) and $\sigma_{\mathrm{IMIM}}$(${}^{15}$N2)] was of −9.66[−14.47] ppm. These findings indicated that each of the building blocks had significantly different magnetic shielding signatures, and so ${}^{15}$N NMR experiment could distinguish the dimeric and tetrameric contents in water.

It should be stressed that the optimized geometry of the IMIM tetramer in gas phase was slightly non-planar, but it had inversion symmetry in the plane with respect to the position of the inner porphyrin ring, that made protonated or unprotonated nitrogen atoms equivalent, as emphasized by the gas phase results in Table 3. In water, the optimized geometry of IMIM obtained with the PCM model was non-planar and exhibited a symmetry break, with an asymmetric distribution of partial charges. The PCM model gave an in-water dipole moment of 2.83 D. Thus, for water-optimized geometry used during the MC simulations, a closer inspection of the water structure around the IMIM tetramer indicated that it occurred an asymmetric distribution of the number of hydrogen bonds. As a result, the ASEC model gave a magnetic shielding difference of 7.16 ppm for the protonated nitrogen atoms. For unprotonated ones the difference was of 4.66 ppm. For comparison, the magnetic shielding differences for protonated [unprotonated] nitrogen ones were of 6.46 [11.12] ppm in HMIM tetramer, which had no inversion symmetry in the plane.

The oligomerization of eumelanin monomers to form dimers and tetramers resulted in significant changes in the magnetic properties. Table 4 shows differences in the magnetic shieldings of nitrogen atoms in dimers and tetramers compared to nitrogen atoms in monomers.

It was found that the magnetic shielding of the protonated nitrogens N1 in HQ and N1 in IQ was further deshielded in HMIM and IMIM. In liquid phase, for instance, the ASEC magnetic shielding differences for N1 in HM, IM, HMIM and IMIM were in the range of −21.42 to −7.15 ppm [−23.70 to 0.29 ppm], relative to the ASEC result of $\sigma_{\mathrm{HQ}}$(${}^{15}$N1) [$\sigma_{\mathrm{IQ}}$(${}^{15}$N1)]. The oligomerization effects could be even more relevant to the magnetic shielding of the unprotonated nitrogen N2 in MQ, which was the further shielded in HMIM and IMIM tetramers. In relation to the result of $\sigma_{\mathrm{MQ}}$(${}^{15}$N2), ASEC magnetic shielding differences for N2 in HM, IM, HMIM and IMIM were in the range of −4.30 to 42.49 ppm. Though comparison with the experiment was not still possible, the theoretical calculations have highlighted that the rather strong sensitivity of the ${}^{15}$N magnetic shieldings with oligomerization may be a helpful tool in determining the structure of eumelanin pigments. Most importantly, in line with the chemical disorder model, the disordered presence of these monomeric and oligomeric building blocks in an eumelanin sample could lead to broadening in NMR features in water, with resonance signals in the spectral range between 78 and 110 ppm and (−7 and −54 ppm) that can be assigned as protonated and (unprotonated) nitrogens in different chemical environments. The intensity of a broad peak is dependent upon the concentration of the species producing that peak.

For a better comparison with the experiment, the theoretical ${}^{15}N$ isotropic chemical shifts, $\delta {(}^{15}N)$, were calculated relative to the ${}^{15}N$ isotropic magnetic shielding constant of liquid nitromethane (−135.8 ppm) obtained from Jameson et al. [62]. The chemical shifts calculated with the ASEC model, which includes the polarization effect of the solvent, ranged between −214 and −246 ppm and (−129 and −82 ppm) that could be assigned as protonated and (unprotonated) nitrogens.

### 3.3. Influence of Stacking on the Imim ${}^{15}N$ Nmr Shielding Constants

**Figure 5:**
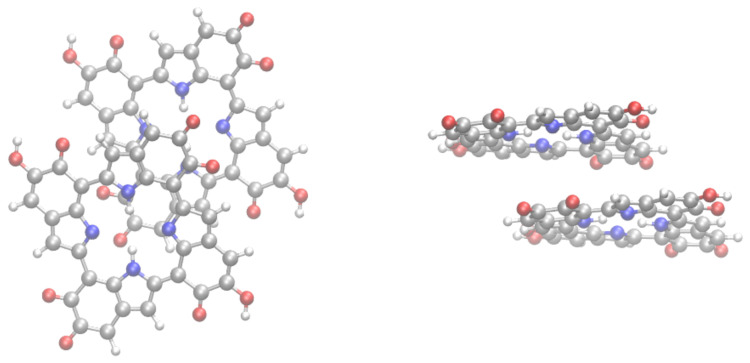
Structure of the IMIM2 dimer. **Left**: top view; **right** (side view).

Several works pointed out the presence of stacked strucures, stabilized by π−π interactions in eumelanin aggregates [21,22,63]. Therefore, it is important to assess how stacking influences the ${}^{15}$N NMR shielding constants in eumelanin. Here, we will discuss stacking by carrying out calculations for the dimer (IMIM2) of the IMIM tetramer. It should be observed that in line with calculations for the free base phthalocyanine dimer [64], dispersion interactions should play a fundamental role on the structural organization and energetics of the dimer. Therefore, geometry optimization for IMIM2 was carried out with B3LYP plus the Grimme D3 semiempirical correction for the dispersion energies, and Becke–Johnson damping (D3BJ) [65]. Figure 5 shows the B3LYP-D3/cc-pVDZ optimized structure of the IMIM2 dimer. The structure was a displaced face-to-face π stacked arrangement. A similar structure was also predicted by B3LYP-D3 geometry optimization of the free base phthalocyanine dimer. The displaced face-to-face structure was energetically stabilized by dispersion interactions [64]. Small deviations from planarity of IMIM species in the IMIM2 dimer were observed (see right panel of Figure 5).

The shielding constants for the protonated N atoms in the IMIM2 dimer of the IMIM species were 82.25, 81.61, 82.65, and 81.76 ppm. Comparison with the values for IMIM (80.23) showed a small shielding of protonated N upon dimerization, which was in the 1.38 to 2.42 ppm range. Shielding constants for the unprotoned N atoms were −10.52,−17.65,−14.85, and −9.94 ppm. Comparison with the IMIM buiding block indicated that upon dimerization unprotonated N atoms were deshielded in a −8.99 to −1.28 ppm spectral range. Although the influence of π stacking was limited to the IMIM2 dimer, it was expected that similar effects on the ${}^{15}$N NMR shielding constants were observed in HMIM stacked structures. It could be also important to investigate the dependence of the NMR shielding constants by carrying out calculations for a large number of π-stacked structures. However, this is out of the scope of the present work.

### 3.4. Conclusions

The nitrogen magnetic shielding constant of a selected set of eumelanin building blocks including monomers, dimers, and tetramers in aqueous solution has been studied using the sequential QM/MM methodology in combination with the ASEC model. Results relying on microsolvation models are also reported. By using the ASEC model, it turns out that nitrogen magnetic shieldings can be significantly modified by the liquid environment polarization, with solvent shifts for unprotonated nitrogens between −3.16 to 36.74 ppm and for protonated nitrogens between −16.59 to 4.75 ppm, relative to the gas phase results. There is a noticeable magnetic shielding contrast that results from the oligomerization of eumelanin monomers to form dimers and tetramers, which could help distinguish the eumelanin building blocks in solution. Therefore, the disordered presence of monomeric and oligomeric building blocks in an eumelanin sample could lead to broadening in NMR features in solution, a picture that would be compatible with a chemical disorder model for the eumelanin pigment. Calculations for the effect of π stacking on the NMR ${}^{15}$N shielding constants were carried out for a dimer (IMIM2) of the IMIM building block. The results indicate that a NMR figerprint of π stacking in eumelanin is a deshielding of the unprotonated N atoms, that in the present case [IMIM2] is in the [−8.99 to −1.28] ppm range. The present results clearly stress the importance of carrying out NMR experiments for eumelanin protomolecules in solution. Moreover, the theoretically predicted NMR shifts may be useful as a guideline in these experiments for a better understanding of the complex structure of eumelanin.

## Figures and Tables

**Table 1:** B3LYP/6-311++G(2d,2p) results for ${}^{15}$N shielding constants (in ppm) of HQ, IQ and MQ monomers. Chemical shifts relative to the gas phase are represented in brackets.

Model	$\sigma_{\mathrm{HQ}}$(${}^{15}$N1)	$\sigma_{\mathrm{IQ}}$(${}^{15}$N1)	$\sigma_{\mathrm{MQ}}$(${}^{15}$N2)
Gas phase	114.55	125.27	−86.55
MS-W${}_{4}$	96.15 [−18.40]	101.82 [−23.45]	−65.72 [20.83]
MS-W${}_{4}$+PCM	96.05 [−18.50]	97.08 [−28.19]	−56.68 [29.87]
ASEC	106.46 [−8.09]	108.68 [−16.59]	−49.81 [36.74]
HB+PC	99.99 [−14.56]	99.97 [−25.30]	−52.23 [34.32]

**Table 2:** B3LYP/6-311++G(2d,2p) results for ^15^N shielding constants (in ppm) of HM and IM dimers. Chemical shifts relative to the gas phase are represented in brackets.

**Model**	$\sigma_{\mathrm{HM}}$ **(** ${}^{15}$ **N1)**	$\sigma_{\mathrm{IM}}$ **(** ${}^{15}$ **N1)**	$\sigma_{\mathrm{HM}}$ **(** ${}^{15}$ **N2)**	$\sigma_{\mathrm{IM}}$ **(** ${}^{15}$ **N2)**
Gas phase	99.41	117.46	−59.39	−55.73
MS-W${}_{5}$	93.14 [−6.27]	107.36 [−10.10]	−54.83 [4.56]	−54.61 [1.12]
MS-W${}_{5}$+PCM	94.86 [−4.55]	104.48 [−12.98]	−50.13 [9.26]	−49.27 [6.46]
ASEC	99.31 [−0.10]	108.97 [−8.49]	−54.11 [5.28]	−51.21 [4.52]

**Table 3:** B3LYP/6-311++G(2d,2p) results for ^15^N shielding constants (in ppm) of HMIM and IMIM tetramers. Chemical shifts relative to the gas phase are represented in brackets.

**Method**	**$\sigma_{\mathrm{HMIM}}$(${}^{15}$N1)**	**$\sigma_{\mathrm{HMIM}}$(${}^{15}$N3)**	**$\sigma_{\mathrm{IMIM}}$(${}^{15}$N1)**	**$\sigma_{\mathrm{IMIM}}$(${}^{15}$N3)**
Gas phase	83.94	91.68	80.23	80.23
MS-W${}_{8}$	90.20 [6.26]	82.22 [−9.46]	82.39 [2.16]	80.46 [0.23]
MS-W${}_{8}$+PCM	83.40 [−0.54]	88.56 [−3.12]	79.49 [−0.74]	78.18 [−2.05]
ASEC	85.04 [1.1]	91.50 [−0.18]	77.82 [−2.41]	84.98 [4.75]
	**$\sigma_{\mathrm{HMIM}}$(${}^{15}$N2)**	**$\sigma_{\mathrm{HMIM}}$(${}^{15}$N4)**	**$\sigma_{\mathrm{IMIM}}$(${}^{15}$N2)**	**$\sigma_{\mathrm{IMIM}}$(${}^{15}$N4)**
Gas phase	−23.30	−24.43	−8.66	−8.66
MS-W${}_{8}$	−22.71 [0.59]	−24.28 [0.15]	−11.41 [−2.75]	−8.56 [0.10]
MS-W${}_{8}$+PCM	−18.77 [4.53]	−23.46 [0.97]	−11.54 [−2.88]	−9.65 [−0.99]
ASEC	−26.45 [−3.16]	−15.33 [9.1]	−11.98 [−3.32]	−7.32 [1.34]

**Table 4:** ASEC results for ^15^N shielding magnetic differences (in ppm) of nitrogens in dimers (HM,IM) and in tetramers (HMIM,IMIM) relative to nitrogens in monomers (HQ,IQ,MQ).

**Method**	**$\sigma_{\mathrm{HM}}$(${}^{15}$N1)**−**$\sigma_{\mathrm{HQ}}$(${}^{15}$N1)**	**$\sigma_{\mathrm{HMIM}}$(${}^{15}$N1)**−**$\sigma_{\mathrm{HQ}}$(${}^{15}$N1)**	
Gas phase	−15.14	−30.61	
ASEC	−7.15	−21.42	
	**$\sigma_{\mathrm{IM}}$(${}^{15}$N1)**−**$\sigma_{\mathrm{IQ}}$(${}^{15}$N1)**	**$\sigma_{\mathrm{HMIM}}$(${}^{15}$N3)**−**$\sigma_{\mathrm{IQ}}$(${}^{15}$N1)**	**$\sigma_{\mathrm{IMIM}}$(${}^{15}$N3)**−**$\sigma_{\mathrm{IQ}}$(${}^{15}$N1)**
Gas phase	−7.81	−33.59	−45.04
ASEC	0.29	−17.18	−23.70
	**$\sigma_{\mathrm{HM}}$(${}^{15}$N2)**−**$\sigma_{\mathrm{MQ}}$(${}^{15}$N2)**	**$\sigma_{\mathrm{HMIM}}$(${}^{15}$N2)**−**$\sigma_{\mathrm{MQ}}$(${}^{15}$N2)**	**$\sigma_{\mathrm{HMIM}}$(${}^{15}$N4)**−**$\sigma_{\mathrm{MQ}}$(${}^{15}$N2)**
Gas phase	27.16	63.25	62.12
ASEC	−4.30	23.35	34.48
	**$\sigma_{\mathrm{IM}}$(${}^{15}$N2)**−**$\sigma_{\mathrm{MQ}}$(${}^{15}$N2)**	**$\sigma_{\mathrm{IMIM}}$(${}^{15}$N2)**−**$\sigma_{\mathrm{MQ}}$(${}^{15}$N2)**	**$\sigma_{\mathrm{IMIM}}$(${}^{15}$N4)**−**$\sigma_{\mathrm{MQ}}$(${}^{15}$N2)**
Gas phase	30.82	77.89	77.89
ASEC	−1.40	37.83	42.49
